# Enterprise digitization and marine economic performance: An empirical study of listed enterprises in China’s maritime economy

**DOI:** 10.1371/journal.pone.0311021

**Published:** 2024-10-30

**Authors:** Quanjun Zhang, Jian Chen, Xiangyu Zhang

**Affiliations:** 1 Guangxi Big Data Research Institute, Nanning, Guangxi, China; 2 Department of Geography, University College London, London, England, United Kingdom; 3 Vanbrugh College, University of York, York, England, United Kingdom; University of Malta, MALTA

## Abstract

The scale and connectivity of marine resources make them more complex than land resource management. Although digitization has been recognized as an organizational change process that can effectively improve resource efficiency and enhance network resilience, however, gaps remain in establishing the theoretical links between digitization and marine economic performance. Based on a panel fixed-effects model, this study evaluates the interrelationships and potential mechanisms of different firms with data from annual reports of listed firms in the marine economy in the eastern coastal region of China. The results indicate that there is a ‘U-shaped’ relationship between digitalization and enterprise efficiency in the maritime sector, and significant heterogeneity exists in the characteristics of these enterprises. Notably, firms’ technological innovation capability can modulate the ‘U-shaped’ relationship through the interaction of economies of scale and economies of scope. This paper highlights how digitization mitigates the fragmentation and sectionalization of marine information and addresses the digital overload and productivity paradox that firms may face in the early stages of digitization. The study suggests that institutional diversity shapes resilience. Governments need to promote top-down regulation and industry collaboration, while marine enterprises need to coevolve collaboratively with them through bottom-up internal communication and external interaction to enhance the value chain of marine enterprises.

## 1. Introduction

Forecasts by the Organization for Economic Cooperation and Development suggest that by 2030, the ocean economy’s contribution to global economic value added will have doubled from its 2016 figures, reaching around 2.5 percent of the global total. A 2021 report by the United Nations Conference on Trade and Development estimated that the global marine industry offers $2.5 trillion in export opportunities [1]. And China is playing an increasingly important role in the development of the global maritime economy. To some extent, maritime trade can flank trends in the maritime economy, because maritime trade has a holistic structure, encompassing economics, trade, politics, law, engineering and logistics, and is linked to all other aspects of technical, social and economic activity [2]. According to the UNCTAD’s Handbook of Statistics 2023 [3], Asia, and China in particular, remains the world leader in maritime transport. In 2021, Asian ports, including China’s, handled a significant portion of the world’s seaborne trade, with China playing a pivotal role in the loading and unloading of cargo. The scale of China’s marine economy continues to expand and is higher than the growth rate of the national economy over the same period, reflecting the strong momentum of the local marine economy. This growth is supported by the integration and innovation of digital technologies and data components in areas such as submarine data centers, offshore wind power, and hydrogen energy storage, facilitating a more extensive and profound integration and optimal allocation of marine resources.

However, economic development has been accompanied by a dramatic decline in marine and coastal biodiversity, with cumulative impacts on marine ecosystems [4]. Over the past 50 years, China has lost approximately 53% of its temperate coastal wetlands, 73% of its mangrove swamps, and 80% of its coral reefs [5]. Currently, China’s marine industry is undergoing a transition from rough to intensive and from labor-intensive to technology-intensive. The strength of marine science and technology have not yet been fully transformed into industrial advantages.

Digitization offers opportunities for the sustainable development of the marine economy [6], not only by facilitating enterprise monitoring and data collection, thereby improving its ecological functions and economic benefits, but also by effectively enhancing awareness of ecological resilience. This helps address the cumulative impacts of human activities on the marine environment and the accompanying sources of stress [7].

Marine resources exhibit scale connectivity, suggesting a propensity for interconnected impacts during their development, a trait that distinguishes their exploitation from terrestrial resources. The development of terrestrial resources typically does not impact non-adjacent areas directly. In contrast, marine resource development can interlink different regions through flowing waters and ambiguous boundaries. This complexity adds significant challenges in both temporal and spatial dimensions for identifying and predicting the development and recovery of marine communities [8–10]. Additionally, the uncertainty surrounding these variables, compounded by the high costs of human observation and research of marine environments, hinders direct human perception of changes in marine ecosystems. In the absence of direct observation, the understanding of marine ecosystems and their response to human interventions is not comprehensive enough [11, 12].

Over the past decade, a substantial body of literature has emerged on the impacts of digital transformation on the economy and society, which is highly relevant to the United Nations Sustainable Development Goal 14 (SDG 14). Governments around the world are increasingly pressured to act on SDG 14, thereby enhancing the economic benefits of the sustainable utilization of marine resources. Currently, the advancement of digital technologies has improved the environment, human health, and the entire food chain. Therefore, more comprehensive research is needed to understand the deep impacts of digital transformation on the socio-ecological system. However, there is limited literature on the dynamic process of digitalization at different stages in the marine economy [13]. Different stages of enterprise transformation impact businesses in various ways, resulting in distinct characteristics for each phase of digitalization in terms of the activities involved, challenges faced, and stakeholder responses.

Academic research on the impact of digitization on corporate value primarily focuses on social and economic values. Firstly, regarding corporate social value, some scholars point out that digitization increases the complexity and dependency among relevant participants, enhances the utilization of infrastructure and resources and promotes sustainable operations for stakeholders in the maritime sector [7, 14]. Maritime security increasingly relies on cyber systems, leading to the incorporation of cybersecurity requirements into maritime regulations [15]. As digital transformation progresses within companies, digital enterprises can share data and resources more efficiently. This enhanced collaboration substantially diminishes information asymmetry among entities involved in digital transformation, thereby bolstering transparency and efficiency throughout the industry [16]. A large number of ports and maritime enterprises continue to operate using legacy systems and disparate technologies, posing challenges in developing a unified platform. The interoperability of these systems is critical for an efficient supply chain. However, achieving this integration often necessitates considerable investments in time, resources, and expertise [17].

Secondly, in terms of economic value, most scholars agree that digitization has a positive impact on firm value. Research have shown that digitalization can effectively shorten the information transmission distance and accurately perceive crisis events [18], thus enhancing the risk management capability and organizational resilience of enterprises [19]. Meanwhile, digitization triggers changes that can break the traditional organizational structure, blur organizational boundaries, give full play to the maximum effectiveness of various resources, and improve the efficiency of enterprise resource utilization [20]. Heikkilä et al. [21] outlined alternatives for the future of smart ports, arguing that digital transformation will be a digital innovation based on the pillars of automation, sustainability, and collaboration as priorities. Digital transformation of enterprises can alleviate financing constraints to some extent. Particularly with the acceleration of digital processes, it can more effectively promote the construction and development of credit systems [22]. This can help alleviate the problems of adverse selection and moral hazard that are common in bank lending, making banks more willing to lend to enterprises. In this way, digitization not only optimizes the internal management of firms but also improves their interaction with financial institutions, which brings more financing opportunities and possibilities to firms [23].

Digitization is driving the marine economy beyond its traditional boundaries and providing numerous new opportunities to enhance productivity, efficiency and sustainability. However, academic and practical research on this topic is still in its early stages, with a significant amount of digitization research being applied to the management of land-based resources or focusing on a specific area, such as the shipping industry. This paper aims to investigate whether digitalization and new technologies can help to make the overall marine economy a more sustainable model. Specifically, it seeks to explore how technological innovation activities in the digitalization of marine enterprises impact corporate performance and to provide theoretical guidance at the strategic level for marine economy enterprises to overcome the negative effects of digitalization. This paper propose an overarching theoretical model that empirically illustrates the U-shaped relationship between corporate digitization and the profitability of marine enterprises. In this model, the introduction of technological innovation is considered a moderating factor and the heterogeneity between state-owned and non-state-owned enterprises (SOEs and non-SOEs) in the context of digitalization in China is clarified.

The remainder of this paper is structured as follows. The second part presents the theoretical mechanisms and research hypotheses based on the review of relevant literature, the third part constructs and describes the research design, including sources of data, selection of variables and model construction. The fourth part comprises empirical testing and results, and the final part discusses implications and insights.

## 2. Theoretical analysis and research hypothesis

### 2.1 Impact of digitization on enterprise performance in marine economy enterprises

Enterprise digitization can be divided into four stages [24–26]. The initial stage of digital transformation involves the construction of the digital infrastructure, including the installation of hardware, the establishment of a network, and the implementation of basic software systems. This serves to establish a robust foundation for the subsequent stages of digitization. The second stage of the process entails integrating internal data resources for centralized management and sharing, employing technologies such as data warehouses and data lakes. In the third stage, digital technologies are employed to optimize and re-engineer business processes. Systems such as ERP and CRM enhance operational efficiency and achieve automation and intelligence. The fourth stage involves the extensive application of advanced technologies, including artificial intelligence, big data analytics, and the Internet of Things. These technologies facilitate business intelligence through predictive analytics, smart manufacturing, and personalized services.

In the early stages of enterprise digitization, the significant initial investments in digital technologies can place significant financial pressure on firms, particularly small and medium-sized enterprises (SMEs). A study by Mocker et al. [27] highlights the upfront cost challenges associated with digital transformation, including acquiring new technologies, systems integration and workforce training. These investments often have uncertain and delayed returns, placing pressure on a firm’s cash flow and financial stability, which is exacerbated by the uncertainty and scale of connectivity of marine resources. Investing heavily in digitalization from the outset can result in significant opportunity costs, while resources allocated to digital transformation may divert funds from other key areas such as product development, market expansion, or human resources. Matt et al. [28] also argue that the opportunity costs of large-scale digital investments early on are particularly detrimental to firms with limited financial resources and may inhibit other strategic areas of growth. Enterprise digitization can be characterized by digital overload and a productivity paradox. The learning and mastery of ever-changing and newly added digital tools can, in itself, increase the complexity and workload of the job. Smith et al. [29] state that the introduction of new digital tools increases the complexity of employee tasks, which leads to confusion and reduced efficiency. The difficulty in training staff and the incompatibility between old and new systems also impede the development of maritime economic enterprises. Fernando [30] posits that many ports have antiquated systems that may be challenging to integrate with new digital solutions, which hinders the collection, sharing, and analysis of data in real-time and in an efficient manner.

Digitization is believed to reduce the cost of running a business, strengthen the division of labor and the level of vertical specialization, and thus increase the efficiency of a company’s output [31–33]. However, As companies continue to digitize, the emergence of digital technologies has the potential to change the relationship between companies’ original production processes, leading to unbalanced and wasteful allocation of resources [34, 35]. As a corollary, if enterprises can promote the synergy of digital technology and the original production process, accelerate the integration of digital technology and the original business process, work mode, and organizational model, it can be considered to achieve cross-business areas, cross-links of the overall optimization of resources, reduce the cost of enterprise operations and thus strengthen the productivity of the enterprise [36]. Agrifoglio et al. [37] posited that the maritime industry can not only expand the scope of digital applications through the integration of information and communication technology (ICT) with production, logistics, sales, and other links, but also integrate the isolated links in the operation and enhance management efficiency.

Current knowledge of the structure and functioning of marine ecosystems is relatively limited for many reasons, including the logistical problems of observing and studying such environments, the high costs associated with them, and the fact that human beings are predominantly terrestrial species. The complexity and variability of the marine environment, in addition to the lack of human expertise in the oceans, are impeding the sustainable development of the marine industry. In response, many marine economic enterprises have begun to introduce digital technologies, such as sensors, into marine fishing, aquaculture, and energy extraction processes with the aim of improving their dynamic monitoring of the marine environment. Many marine economic enterprises have even begun to utilize the results of digital technology monitoring for production decision-making. With the advancement of digital transformation in enterprises, the expansion of digital capabilities has become a key factor in improving the efficiency and productivity of the marine industry. Bharadwaj et al. [38] demonstrated that firms that effectively expand their digital capabilities can significantly reduce operating costs, improve agility, and enhance customer experience. Furthermore, digital transformation facilitates the optimization of corporate organizational structure, the upgrading of human capital, and the enhancement of corporate market influence. This, in turn, leads to the optimization of resource allocation and the improvement of total factor productivity in the marine economy [39, 40].

In conclusion, the digital economy brings with it information resources that incur high storage costs and crowd out large amounts of production resources. Consequently, enterprises require higher-level technological innovation and resource allocation strategies to cope with the complexity and variability of the environment and the uncertainty of technological application when facing instability in the marine economy [41]. Although the initial investment in digitization is considerable and thus affects the efficiency of enterprises, digitization will continue to yield benefits as digitization progresses smoothly.

In light of the preceding analysis, this study proposes the following hypothesis:

**H1:** There is a clear ‘U-shaped’ relationship between enterprise digitization and enterprise performance in the maritime economy.

### 2.2 The moderating role of technological innovation in enterprises

Technological innovation is a significant factor in enterprise digitization. Firms that adopt new technologies, such as big data, artificial intelligence, and cloud computing, are able to optimize their business processes and improve operational efficiency [38]. According to Dynamic Capabilities Theory and Organizational Change Theory, firms must continuously update and reorganize their resources and capabilities to respond to changes in the external environment. Technological innovation enables enterprises to enhance their dynamic capabilities and better adapt to the rapid changes in the digital era, thus maintaining competitiveness and enterprise performance in the market [42].

The introduction of new technologies and the effectiveness of their application by enterprises depend on their ability to rapidly acquire, fully assimilate, and effectively apply these technologies [43]. In light of the rapid advancement of information technology, enterprises with a robust capacity for innovation can enhance the intelligence of their production and operations through algorithm optimization and the application of novel technologies. This, in turn, can facilitate the optimization of data-to-information conversion [44], enabling data-driven information matching analysis, inventory management and product optimization and upgrading. These endeavors not only enhance management efficiency and reduce production costs but also bolster the international competitiveness of enterprises through technological advantages.

Compared to land areas, marine areas arguably tend to support a wider range of uses due to their three-dimensional nature, combined with expectations of freedom of the seas and assumptions about their resilience and productivity. Encouraging innovative organizations can be more effective in dealing with the resistance to change associated with digital transformation efforts and driving the digitalization of the firm [45]. Innovation is closely related to improving problem-solving capabilities. innovative firms utilize their advanced problem-solving skills to improve operational efficiency by leveraging digital tools to address complex challenges. A corporate cultural climate that encourages innovation has been found to improve the ability of organizations to adapt to change, which is critical in integrating digital technologies into existing business processes [46]. In conclusion, the digitization of marine economic enterprises can only truly facilitate the innovative development of enterprises if it is accompanied by the capacity to innovate. Enterprise digitization can provide enterprises with a greater quantity of data and information, but the challenge lies in determining how to utilize these data and information for innovation. Only through the application of technological innovation can enterprises identify problems and opportunities within the context of vast data sets, explore novel business models and product services that are more aligned with the development of the marine economy, and enhance their own benefits.

In light of the aforementioned evidence, Hypothesis 2 is put forth for further investigation in this study:

**H2:** In the context of the marine economy, the introduction of technological innovation can serve to moderate the inverse ‘U-shaped’ relationship between enterprise digitization and enterprise performance.

### 2.3 Heterogeneity of digitization impacts in state-owned enterprises and non-state-owned enterprises

Theory of the resource-based view suggests that an organization’s resources and capabilities are the source of its competitive advantage [47]. Given the diversity of resources and capabilities among organizations, these differences can give rise to disparate organizational change processes, each exhibiting distinct approaches and outcomes [48]. State-owned enterprises (SOEs) typically receive policy and financial support from the government during the process of organizational change, thereby alleviating the pressure of declining profits. Such support may take the form of tax incentives, financial subsidies, and other measures designed to ensure that SOEs remain somewhat profitable during the change period. In contrast, non-SOEs face greater market risks and greater variability in benefits during organizational change [49]. Digital transformation is not merely a technological change. It is a comprehensive organizational change that entails profound alterations in the enterprise’s structure, processes, and culture. Consequently, different tenure firms possess varying resources, which may influence the nature of their digital transformation and its consequences [38].

State-owned enterprises (SOEs) and non-state-owned enterprises (NSOEs) in the Chinese context may adopt different enterprise digitization paths due to factors such as organizational structure, strategic focus, regulatory mechanisms, and corporate culture, which in turn have different impacts on enterprise effectiveness. Firstly, in terms of organizational structure, SOEs often have a large-scale and monopoly nature. Consequently, in the process of implementing the digital transformation plan, they will be affected by the original business inertia of internal departments, as well as by external political aspects [50]. Under the influence of multiple factors, SOEs will prioritize stability and long-term goals over short-term financial gains when choosing a digital transformation model. In contrast, NSOEs are typically more focused on profitability and shareholder value, and therefore prioritize short-term returns on digitalization investments. They are also more susceptible to fluctuations in corporate profits caused by digitization [51, 52].

In terms of strategic focus and the choice of digitization strategy, SOEs may adopt digital technologies for a variety of strategic reasons, such as to improve the quality of public services or to meet specific government objectives. It has been shown that the more competitive the industry, the stronger the contribution of digital transformation to business resilience [53]. These measures do not necessarily lead directly to efficiency gains. In contrast, NSOEs tend to be more inclined to closely align their digitalization strategies with efficiency gains and competitive advantage [54, 55]. In contrast, SOEs operate within a regulatory framework that is heavily influenced by government regulations, which can act as both a barrier and an enabler of digitalization. In contrast, NSOEs can adopt more flexible digitization strategies in the face of fierce competition, thereby stabilizing profit levels [56].

Finally, with regard to organizational culture, there are notable differences between SOEs and NSOEs. For instance, SOEs tend to exhibit a greater aversion to risk, whereas NSOEs are more inclined to embrace risk-taking. These cultural differences have a significant impact on the role of digitization in enhancing the efficiency of their respective firms [57]. In light of these observations, this study proposes Hypothesis 3.

In light of these observations, this study proposes Hypothesis 3.

**H3:** There is considerable heterogeneity in the ‘U-shaped’ relationship between enterprise digitization and enterprise performance among SOEs and NSOEs enterprises in the marine economy.

**Fig 1:**
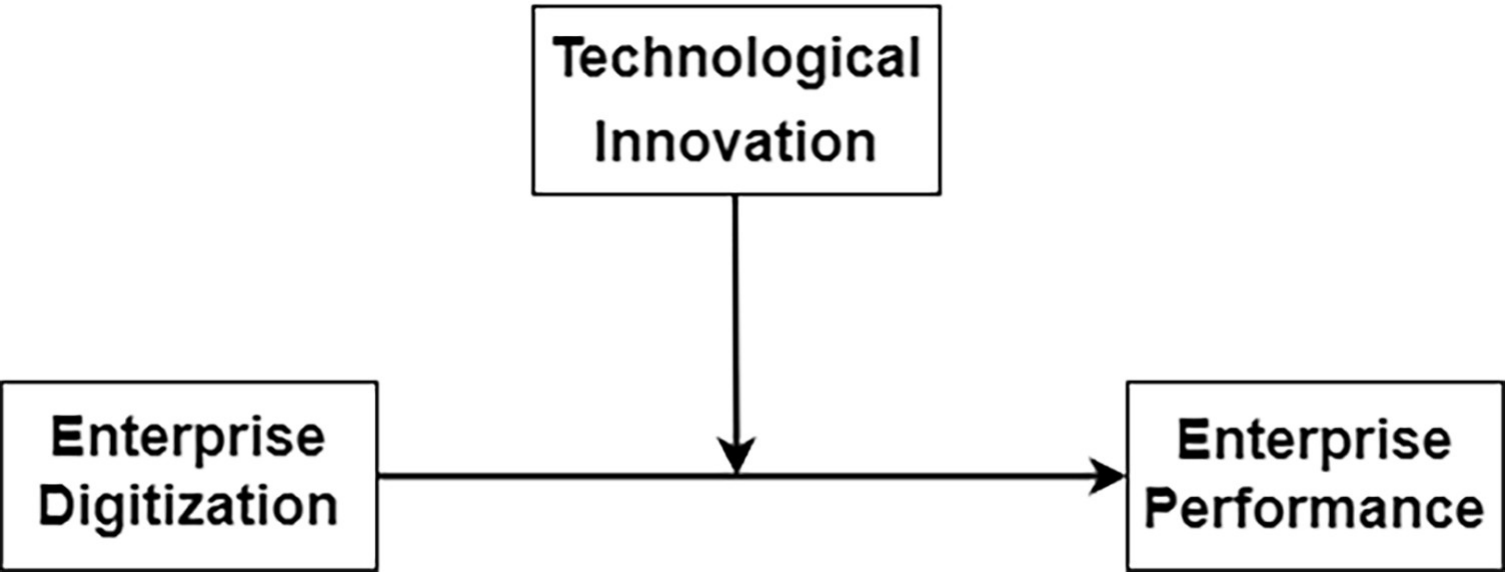
Theoretical model.

In conclusion, the present theoretical model is constructed as illustrated in **Fig 1.**

## 3. Research design

### 3.1 Sample selection

The blue economy is the term used to describe economic activities related to the oceans. The World Bank defines the blue economy as ‘sustainable use of ocean resources to benefit economies, livelihoods and ocean ecosystem health’ [58]. For this study, corporate annual reports of 108 publicly traded companies from 2016 to 2020 were analyzed, adhering to this definition and reflecting the relevant policies and plans of China. In the analysis of data from publicly listed companies, it is standard practice to exclude stocks labeled as special treatment (ST) and particular transfer (PT). This is primarily because these labels indicate that the companies are facing financial risks, and may be at risk of delisting. Such conditions can affect the accuracy and general applicability of data analysis [59]. After excluding companies under ST and PT designations, those delisted during the study period, and those with missing key data, a dataset of 536 valid observations was compiled. The enterprises included in this study are located in ten provinces along the eastern seaboard of China, including Guangdong, Guangxi, Hainan, Liaoning and Shandong, spanning diverse industries such as water transportation and fisheries.

### 3.2 Definition and measurement of variables

The independent variable is enterprise digitization (Di for short). Current research on the measurement of enterprise digitization focuses on two approaches. The first approach is to construct an indicator system to carry out scoring. For example, scholars such as Zhao [14], Pan and Gao [60] constructed an indicator system to measure the level of enterprise digitization from four key areas: digital technology application, internet business model, intelligent manufacturing and modern information system. The second method employed text mining to ascertain the frequency of keywords related to digitization in the annual reports of sample enterprises, as exemplified by Brodny & Tutak [61]. The construction of an indicator system score necessitates the availability of a substantial quantity of data, rendering its application to all types of enterprises challenging. In order to facilitate data acquisition and enhance objectivity, this study employed Python to collate word frequencies pertaining to ‘enterprise digitization’ within the annual reports of listed companies operating within the ocean economy. The collated word frequencies were then converted into logarithms, which were subsequently utilized to calculate the level of enterprise digitization.

The dependent variable is enterprise performance (EP). Return on Total Assets (ROA) and Rate of Return on Common Stockholders’ Equity (ROE) are frequently employed as proxy variables for firm performance [62]. Brealey et al. [63] posited that Return on Assets (ROA) is the fundamental financial ratio utilized to assess the financial performance of a firm. ROA is widely recognized as an important indicator of overall operational efficiency and profitability of a firm. It is calculated by dividing net profit by total assets and provides a valuable indicator for assessing the efficiency of a firm’s asset utilization. The trajectory of ROA can serve as an early indicator of potential future business risk. Should ROA continue to decline, it may indicate that a firm’s asset utilization efficiency is declining or that the firm is facing greater business risk [64]. This study therefore proposes the use of return on total assets (ROA) to assess business performance.

The moderating variable in this study is the firm’s scientific and technological innovation (IN). At present, academics employ three principal methodologies for gauging corporate technological innovation. The first is research and development (R&D) expenditures [65], the second is the number of patents a firm has, and the third is new product announcements [66]. In summary, patents are a reflection of actual innovations, whereas R&D expenditures represent only inputs. It is possible that R&D expenditures may not result in innovations, or that these inputs may be used for other purposes (e.g., to improve an existing product rather than to develop a new one) [67]. Concurrently, the data pertaining to R&D expenditures may not be entirely transparent or consistent due to the differing accounting standards employed by firms or for reasons of confidentiality [68]. Finally, patents may encompass a diverse array of innovation types, including product, process, and design innovations. Conversely, research and development (R&D) expenditures may be concentrated on specific R&D projects or areas, and may not fully reflect the firm’s overall innovation capability [69]. In light of the aforementioned considerations, this study employs patent applications as a means of gauging the extent of technological innovation exhibited by enterprises.

To ensure a comprehensive reflection of the objective phenomena and to mitigate the risk of omitting critical variables that could lead to testing errors, this study incorporates both micro and macro factors as control variables. Given that the operation of enterprises is significantly influenced by the external economic environment, this study includes the value added of the gross domestic product (GDP, measured in billions of CNY) as a macroeconomic control variable. Vial [70] highlighted that older companies might face greater organizational and cultural challenges in adapting to digital changes. Additionally, Tang et al. [71] demonstrated that independent directors play a crucial role in fostering technological innovation and transformation within firms. Consequently, this study also incorporates firm age (Age) and the percentage of independent directors (Rind) as micro-level control variables. Moreover, enterprise digitization requires substantial upgrades to technical equipment, often necessitating significant fixed asset investments. A higher ratio of fixed asset investment may indicate that firms have the resources needed for technological upgrades essential for firm digitalization. Hagiu and colleagues [72] suggested that greater technology investment allows firms to secure a first-mover advantage in digital transformation, thereby enhancing their market competitiveness and profitability. Echoing this, Brynjolfsson and McElheran [73] argued that such investments could significantly improve firm profitability. Therefore, this study includes the ratio of total investment in fixed assets to total assets (FA) and investment in science and technology R&D (TI) as additional control variables.

### 3.3 Data source

The primary data sources discussed in this paper are databases from the United Nations Conference on Trade and Development (UNCTAD) and the National Bureau of Statistics of China (NBS), focusing on the GDP of China’s coastal provinces and fixed asset investment in these areas. We also utilized key data points from corporate annual reports obtained from the Wind and CSMAR databases. These reports were analyzed by a third-party company employing big data tools, including analyses of word frequency in annual reports, financial statement data and patent data of enterprises. The timeframe for the data used in this study spans from 2016 to 2020. A descriptive analysis of the collected data is presented in **Table 1**.

**Table 1:** Descriptive statistics.

Variable type	Variable	Count	Mean	SD	Min	Max
Dependent variable	EP	536	0.028	0.165	-3.164	0.202
Independent variables	Di	536	2.438	0.959	0.000	4.997
	Di^2^	536	6.860	4.816	0.000	24.972
Control variables	GDP	536	71973.070	27947.522	4053.200	110760.940
	Age	536	12.441	7.585	-4.000	28.000
	Rind	536	0.376	0.067	0.250	0.700
	FA	536	0.334	0.167	0.005	0.805
	TI	536	5.773	0.876	2.523	7.064
Moderating variable	IN	536	31.115	62.744	0.000	573.000

### 3.4 Model setting

A variety of nonlinear functions, including quadratic, exponential, and other functions, can be used to model U-shaped relationships. Among these, the most common approach for verifying U-shaped relationships is to add a squared term to a quadratic function [74]. In order to test the mechanism of enterprise digitization on the performance of enterprises in the marine economy, this study first establishes the following model, as shown in **Eq (1)**.


$${EP}_{it}=\beta_{0}+\beta_{1}{Di}_{it}+\beta_{2}{Di}_{it}^{2}+{\lambda Control}_{it}+\mu_{i}+\nu_{t}+\varepsilon_{it}$$ (1)

In this context, the subscripts i and t represent the sample unit and time, respectively. The independent variable *Di* represents the enterprise digitization. The dependent variable *EP* represents the enterprise performance. *Control* represents the control variables, including the GDP of the province where the enterprise is located *(GDP)*, firm age *(Age)*, the percentage of independent directors *(Rind)*, assets to total assets *(FA) and* investment in science and technology R&D *(TI)*. The subscript *μ*_*i*_ denotes the individual fixed effect, while the subscript *ν*_*t*_ denotes the time—fixed effect. The comprehensive effect of enterprise digitization and enterprise performance on the marine economy is determined by testing the coefficients *β*_*1*_ and *β*_*2*_ of the core variables of the baseline regression Eq (1) to ascertain whether they are significant at the confidence level.

In order to further test the moderating effect of enterprise technological innovation on the relationship between enterprise digitization and enterprise performance, this study establishes a moderating effect model based on the benchmark model, as shown in **Eq (2)**. Where, ${Di}_{it}\times {IN}_{it}$ denotes the interaction term of enterprise digitization and technological innovation. ${Di}_{it}^{2}\times {IN}_{it}$ denotes the interaction term of enterprise digitization and technological innovation.

$${EP}_{it}=\beta_{0}+\beta_{1}{Di}_{it}+\beta_{2}{Di}_{it}^{2}+\beta_{3}{Di}_{it}\times {IN}_{it}+\beta_{4}{Di}_{it}^{2}\times {IN}_{it}+{\lambda Control}_{it}+\mu_{i}+\nu_{t}+\varepsilon_{it}$$ (2)

This study conducts regression analysis and tests for moderating effects based on the aforementioned model.

## 4. Empirical test and analysis

### 4.1 Results of basic regression analysis

This study employs STATA 15 to conduct the test and data analysis is conducted using data from listed companies in the marine economy of China’s coastal provinces from 2016 to 2020. Initially, this study examined the variance inflation factor (VIF) to identify the presence of multicollinearity in the model variables. The results indicate that the variance inflation factor (VIF) values of all variables are less than 2, which is much lower than the commonly used critical value of 10. This suggests that there is no significant multicollinearity problem in the model. Secondly, to determine whether there is an autocorrelation problem in the model, this study applied the Durbin-Watson (DW) test. The results of the test indicate that autocorrelation is not significant in the model. The decision between a fixed effects model and a random effects model is a pivotal step in the analysis of panel data. The results of the Hausman test indicate that the fixed effects model is more appropriate for the data structure of this study. The regression analysis results are displayed in **Table 2**, aiming to ascertain if a nonlinear relationship exists between the level of enterprise digitization and enterprise performance.

**Table 2:** Basic regression results.

	(1)	(2)	(3)	(4)
Di	-0.0028**	-0.0198**	-0.0336**	-0.0304**
	(-0.1626)	(-0.4457)	(-1.2020)	(-1.0706)
Di^2^	0.0021**	0.0053**	0.0043**	0.0043**
	(0.1218)	(0.5375)	(0.7195)	(0.7203)
GDP		-0.0021**	-0.0023***	-0.0018**
		(-1.0084)	(-1.1877)	(-1.2589)
Age		0.0090**	-0.0095	-0.0098**
		(0.7953)	(-1.5063)	(-1.5431)
Rind		0.0970	0.0651	0.0617
		(0.6064)	(0.6703)	(0.6340)
FA		0.4515***	-0.2195***	-0.2223***
		(4.1228)	(-3.1374)	(-3.1704)
TI		0.1367**	0.0701**	0.0704
		(1.6589)	(1.3105)	(1.3152)
IN			-0.0001**	0.0001**
			(-0.3306)	(0.3859)
Di×IN			-0.0025**	-0.0014**
			(-0.8382)	(-0.4276)
Di^2^×IN				0.0035**
				(0.7357)
cons	0.0369	-0.8877**	-0.0944	-0.0917
	(0.9001)	(-1.7234)	(-0.2847)	(-0.2764)
id	Yes	Yes	Yes	Yes
year	Yes	Yes	Yes	Yes
*N*	536	536	536	536
R^2^	0.0272	0.0718	0.0864	0.0878

*t* statistics in parentheses

** *p* < 0.05

*** *p* < 0.01

This objective is pursued by analyzing both the primary and the square term of enterprise digitization in the model (1). Findings from the model (1) indicate a negative impact of enterprise digitization on performance (β = -0.0028, p < 0.05), complemented by a positive, significant impact of the square term of enterprise digitization on performance (β = 0.0021, p < 0.05). Model (4) demonstrates that the coefficient for enterprise digitization remains negative and statistically significant (p < 0.05), while the coefficient for the square term of enterprise digitization is positive and also significant (p < 0.05), confirming a ‘U-shaped’ relationship between the digitization level and performance. This pattern suggests that enterprises may initially see a decline in profitability and value at the onset of digitization efforts. Still, as digitization deepens, substantial benefits accrue, thus forming a ‘U-shaped’ curve. Accordingly, **H1** is supported.

Enterprise digitization generally requires significant initial investments, including the acquisition of new technological equipment, software, and systems. Additional investments in infrastructure and data security systems are often necessary, which can increase operational costs during the initial stages of digitization. However, as the digitization unfolds, the long-term benefits become increasingly apparent. These benefits include enhanced operational efficiency, improved data analytics, and an enriched customer experience, collectively contributing to a rise in enterprise profitability.

In recent years, digitization, propelled by technological innovation, has become increasingly prevalent and is accelerating. Technological innovation involves not only the adoption of existing technologies but also the enhancement and novel application of these technologies. Such innovative approaches can tailor digital tools and solutions more effectively to the specific operational models and business needs of marine enterprises, thereby enhancing the effectiveness of digital implementation and improving enterprise performance. Accordingly, this study identifies enterprise technological innovation as a moderating variable and investigates its moderating effect across the ‘U-shape’ curve. The findings are detailed in **Table 2**. The analysis within models (3) and (4) examines the nonlinear moderating role of enterprise science and technology innovation in the relationship between enterprise digitization and performance. It reveals that the coefficient for the interaction term between enterprise science and technology innovation and digitization is -0.0014, which is statistically significant at the statistical level of 5%.

The coefficient for the interaction term between enterprise technological innovation and the square term of enterprise digitization is 0.0035, which is statistically significant at the statistical level of 5%. This finding indicates that enterprise technological innovation exerts a ‘U-shaped’ moderating effect on the relationship between digitization and enterprise efficiency. Initially, there is a negative moderating effect when firms engage in technological innovation, which may lead to increased investment in marketing due to the initially higher costs compared to the benefits. However, although the initial investments in digitization and technological innovation can be substantial and may impact short-term profitability, continuous innovation and optimization enable the enterprise to sustain a competitive market advantage and achieve long-term cost-effectiveness and revenue maximization. Over time, the investments in technological innovation and digitization transform into significant enterprise revenue, thereby exerting a positive moderating effect. These results robustly support the **H2**.

### 4.2 Robustness test

This study utilizes a fixed effects model to control for individual characteristics and temporal variations. Despite this, there may still be pronounced time trends or cyclical fluctuations within the data that could influence the accuracy of the model’s estimates. To assess the model’s robustness across different economic conditions, this study proposes to follow the approach of James et al. [75] by narrowing the sample timeframe to the 2019–2020 period. This adjustment aims to mitigate the potential impact of long-term trends on the model’s outcomes. The results of model (4) in **Table 3** indicate that, after controlling for relevant variables, enterprise digitization and its interaction term significantly influence enterprise performance, with digitization showing a negative effect (β = -0.0315, p < 0.05) and its interaction term demonstrating a positive effect (β = 0.0077, p < 0.05). In the meantime, the squared term of enterprise digitization (β = 0.0029, p < 0.05) and the interaction term between the squared term and IN (β = 0.0002, p < 0.05) demonstrated a significant positive effect on enterprise performance. This indicates that hypotheses 1, 2, and 3 are valid with a reduced sample. Consequently, the results of the modeling analysis of this study have passed the robustness test.

**Table 3:** Robustness test.

	(1)	(2)	(3)	(4)
Di	-0.0075**	-0.0077**	-0.0253**	-0.0315**
	(-0.3361)	(-0.1035)	(-0.5217)	(-0.6431)
Di^2^	0.0050**	0.0027**	0.0027**	0.0029**
	(0.2230)	(0.1803)	(0.3138)	(0.3392)
GDP		-0.0001	-0.0000	-0.0000
		(-1.0569)	(-1.1451)	(-1.3086)
Age		-0.0053**	-0.0045***	-0.0046***
		(-1.3294)	(-3.2299)	(-3.2985)
Rind		-0.1079	-0.1334	-0.1117
		(-0.4857)	(-0.9164)	(-0.7544)
FA		-0.0026	-0.0053	-0.0012
		(-0.0187)	(-0.0757)	(-0.0179)
TI		0.1115	0.0109	0.0177
		(1.6346)	(0.3304)	(0.5187)
IN			0.0001**	-0.0004**
			(0.0053)	(-0.7578)
Di×IN			0.0054**	0.0077**
			(1.2898)	(1.5367)
Di^2^×IN				0.0002**
				(0.8306)
cons		-0.4019	0.1920	0.1689
		(-1.2109)	(1.2203)	(1.0565)
id		Yes	Yes	Yes
year		Yes	Yes	Yes
*N*		213	213	213
R^2^		0.0336	0.0048	0.0058

*t* statistics in parentheses

** *p* < 0.05

*** *p* < 0.01

### 4.3 Heterogeneity test

Although our analysis identified a nonlinear relationship between enterprise digitization and performance, it remains unclear whether this effect varies across different types of enterprise ownership. Consequently, further investigation is warranted. In the context of Chinese culture, SOEs and NSOEs differ significantly in financial and human resources, as well as in governance structures. Following the methodology of Song et al. [76], this study categorizes the sample into SOEs and NSOEs and conducts separate regression analyses for each group. The results, presented in **Table 4**, indicate that the digitization coefficient for SOEs (-0.0468) is smaller than that for NSOEs (-0.0072), with both coefficients being statistically significant at the statistical level of 5%. This finding suggests that in the initial stages of digitization, the profitability of NSOEs declines more rapidly than that of SOEs. The introduction of digital technologies can entail substantial costs, including the acquisition of technical equipment, system upgrades, and staff training. Due to their robust financial backing and government support, SOEs are better equipped to withstand these early-stage risks, resulting in a lesser decline in efficiency compared to NSOEs. Private enterprises, lacking similar financial and resource capacities, may experience a more rapid decline in enterprise value due to the immediate financial burden of digitization investments. However, as digitization deepens and performance improves, the inherent advantages in scale and resources of SOEs become increasingly apparent, as highlighted by Chen & Wu [77], leading to greater scale efficiency compared to their private counterparts. Thus, **H3** of this study is substantiated.

**Table 4:** Heterogeneity test.

	SOEs	NSOEs
Di	-0.0468**	-0.0072**
	(-0.5381)	(-0.4249)
Di^2^	0.0138**	0.0045**
	(0.7472)	(1.3025)
GDP	0.0001	-0.0001***
	(0.0380)	(-2.6315)
Age	-0.0006**	-0.0040***
	(-0.1624)	(-4.6828)
Rind	-0.2961	0.0506
	(-1.0406)	(0.7684)
FA	0.3769***	-0.1369***
	(2.9272)	(-3.6563)
TI	0.0377**	0.0172**
	(0.9830)	(1.2976)
cons	-0.2083**	0.0922**
	(-0.9828)	(1.3986)
*N*	214	322
R^2^	0.0880	0.1394

*t* statistics in parentheses

* *p* < 0.1

** *p* < 0.05

*** *p* < 0.01

## 5. Discussion and conclusion

### 5.1 Discussion

This study employs panel data from annual reports and other annual reports of listed companies in the marine economy industry in China’s coastal provinces during the period from 2016 to 2020. The nonlinear U-shaped relationship between digitization and enterprise efficiency of marine economy enterprises is empirically tested. The moderating effect of enterprise science and technology innovation on the aforementioned nonlinear U-shaped relationship is analyzed using the moderating effect model. The study also examines whether there is heterogeneity in the impact of enterprise digitization on enterprise efficiency between state-owned and non-state-owned enterprises in the context of the marine economy. This study provides an in-depth analysis of the important role of enterprise digitization in the marine economy in inhibiting and then promoting enterprise efficiency. Finally, the data analysis results of this study support **H1**, **H2** and **H3**. The detailed conclusion and discussion are presented:

This study identifies a ‘U-shaped’ relationship between enterprise digitization and performance in the marine sector. In the initial stages of digitization, enterprises face substantial investments in capital, human resources, and time to develop and deploy digital infrastructures and systems. Moreover, employee training to adapt to these new systems may temporarily impair work efficiency, leading to higher digitization costs and potentially reduced benefits. This is analogous to the findings of Kilimis et al. [78], who conducted research on German firms and discovered that the implementation of digitization is still gradual, particularly for SMEs. These enterprises tend to misunderstand the intricacies and expenses associated with digitization, which may result in a high initial investment in digitization that could compromise the profitability of the firm. Over time, however, the advantages of digitization in terms of efficiency and productivity become increasingly evident. Optimized data management and smarter decision-making, fueled by big data analytics, enhance the effectiveness of business operations. The integration of digital technologies also streamlines operational processes and cuts costs. In a similar vein, Soroka et al. [79] also discovered that digital analytics tools can assist SMEs in measuring insights and optimizing existing business processes. This, in turn, reduces operational costs and improves business performance. Enterprises that deeply embed digital technologies can exploit data and technological innovations to devise new business models and generate additional revenue streams. For example, the creation of smart fisheries systems using the internet of things (IoT) and web technologies facilitates seamless interaction and interoperability along the fisheries chain. This allows for the effective alignment of resources from various sectors with the needs of the fishery industry, optimizing the use of fishery resources and enhancing operational efficiency and profitability. Moreover, through rigorous data collection, fishery enterprises can gain real-time, accurate insights into the marine environment, predict the status of biological resources, and determine optimal fishing methods and timing. As digitalization deepens and matures, enterprises achieve heightened operational efficiency, enhanced market adaptability, and sustained innovation, culminating in notable improvements in profitability and operational performance. Guan and Li [80] pointed out that the digital economy promotes the high-quality development of the marine economy and its spatial spillover effects. The results of this study support this thesis.

Technological innovation in enterprises moderates the ‘U-shaped’ relationship between enterprise digitization and enterprise performance. Technological innovation includes not only the technology itself, but also the innovation of work processes, management practices, and business models [81]. The impact of the digital economy on marine enterprises is predominantly manifested through the interplay between economies of scale and economies of scope. Technological innovation enables these enterprises to enhance efficiency, reduce costs, develop new markets and augment the value of their products and services, thereby accelerating the return on investment. For instance, in storm surge monitoring, leveraging marine big data integrated with coastal city information enhances the capabilities of early warning systems, disaster prevention and mitigation, and disaster assessment through comprehensive big data analysis and mining [82]. In pelagic fishing, the integration of marine big data with vessel location data, operational information, and fishing forecasts allows for informed decision-making prior to fishing activities, optimizing the utilization and potential of pelagic fishing resources [83]. Similarly, in oil spill monitoring, combining marine big data with ship traffic and port channel information facilitates detailed analysis of oil spill patterns and characteristics. Furthermore, the digital economy empowers these enterprises to utilize their existing customer base to expand into a variety of other businesses at minimal cost, thus realizing business expansion and profit growth through economies of scale. This strategic approach not only streamlines operations but also significantly enhances market responsiveness and financial performance.

This study of heterogeneity revealed that the impact of digitization on business efficiency in SOEs within the marine economy differed from that of non-SOEs. This is mainly reflected in the fact that in the pre-digitization period, the decline in the benefits of non-SOEs was more pronounced than that of SOEs. However, after firms enter the deeper stage of digitization, SOEs’ benefits rebound faster than those of non-SOEs. This result is consistent with the findings of Lisdiono et al. [84], who observed that SOEs possess greater capacity to operate information technology and collect analytical capabilities during periods of organizational change, leading to enhanced organizational resilience. The initial phase of digital technology adoption often necessitates significant investment in high-end technological equipment, system upgrades, and employee training. In the initial stage of digitization, SOEs are typically better able to withstand the associated risks and experience a lower decline in corporate efficiency than non-SOEs due to their strong financial strength, government support, and overall strength. Wang et al. [85] also indicate that SOEs receive more financial support from the government than do non-SOEs, thereby alleviating their financial pressures. In contrast, non-state nonprofit organizations may lack sufficient funds and resources to cope with the financial pressures associated with these initial investments. As the digitalization process continues to advance, the scale and resource advantages of SOEs gradually emerge, giving them an advantage in the digital transformation process. Therefore, this study serves as a corroboration and supplement to the studies of scholars.

### 5.2 Conclusion

On the basis of the above empirical research and theoretical analysis, this study makes the following conclusion based on the findings of the study.

First, data, as the most critical production factor in the digital economy, can effectively mitigate the issues of fragmentation and departmentalization in ocean-related information, reducing delays and asymmetries in information circulation. Central government could foster synergistic progress in the inter-regional marine industry through a top-down approach, transforming it from a resource-dependent to a new type of industry based on management innovation, scientific advancement and workforce quality improvement. This transformation will promote cooperative integration across the industrial chain in aspects such as supply, data, technology, and production modes. Enterprises likewise need to strengthen cooperation with key digital economy industrial parks under top-down guidance to enhance the scale and intensity of the development of the marine economy, extend the industrial chain, cultivate emerging marine industry clusters and promote the upgrading of the value chain.

Secondly, social capital could participate in the digital transformation of the marine economy in a bottom-up manner. The hierarchical structure of the past is ill-suited to today’s dynamic, complex, and uncertain market environment. Digital transformation enables local governments and enterprises to improve the efficiency of internal communication and external interaction, enabling organizations to more effectively access complex local information. The organizational structure of many NSOEs in the marine field has gradually tended to adopt a platform model because they must adopt more competitive market strategies and blur organizational boundaries to improve environmental adaptability. Given the high initial costs of digitization, local governments could implement tailored policies to encourage social capital to participate in marine governance from the bottom up, strengthen regulatory system construction and support digital transformation. Collaborative development of supply chain finance among different enterprises helps financial institutions provide services such as accounts receivable pledge loans, alleviating financing difficulties for SMEs. Furthermore, venture capital and angel investment can mobilize idle funds to support enterprise digital transformation.

Finally, systems coevolve through interactions between top-down and bottom-up processes. Lower levels may better understand governance efficiency but may lack a comprehensive perspective on ecological and sustainability issues. Therefore, it is crucial to ensure synergistic actions across different levels to address broader challenges strategically. When providing public services such as technical support for digital transformation, data resource sharing, business guidance, industry incubation and research planning, the government could tailor strategies to the specific characteristics of various marine enterprises, fostering local development and generating a batch of replicable and scalable experiences to systematically promote integrated development. This requires the coevolution of top-down and bottom-up approaches, alongside market-driven, collective learning and communication strategies to create synergistic effects that leverage the strengths of one approach to offset the weaknesses of another [86].
